# MAIT Cell Recognition of MR1 on Bacterially Infected and Uninfected Cells

**DOI:** 10.1371/journal.pone.0053789

**Published:** 2013-01-14

**Authors:** Mary H. Young, Lance U’Ren, Shouxiong Huang, Thierry Mallevaey, James Scott-Browne, Frances Crawford, Olivier Lantz, Ted H. Hansen, John Kappler, Philippa Marrack, Laurent Gapin

**Affiliations:** 1 Integrated Department of Immunology, National Jewish Health and University of Colorado School of Medicine, Denver, Colorado, United States of America; 2 Howard Hughes Medical Institute, University of Colorado School of Medicine, Aurora, Colorado, United States of America; 3 Department of Medicine, University of Colorado School of Medicine, Aurora, Colorado, United States of America; 4 Department of Pharmacology, University of Colorado School of Medicine, Aurora, Colorado, United States of America; 5 Program in Biomolecular Structure, University of Colorado School of Medicine, Aurora, Colorado, United States of America; 6 Department of Biochemistry and Molecular Genetics, University of Colorado School of Medicine, Aurora, Colorado, United States of America; 7 Inserm Unit 732, Institut Curie, Paris, France; 8 Department of Pathology and Immunology, Washington University School of Medicine, St. Louis, Missouri, United States of America; The Ohio State University, United States of America

## Abstract

Mucosal-associated invariant T cells are a unique population of T cells that express a semi-invariant αβ TCR and are restricted by the MHC class I-related molecule MR1. MAIT cells recognize uncharacterized ligand(s) presented by MR1 through the cognate interaction between their TCR and MR1. To understand how the MAIT TCR recognizes MR1 at the surface of APCs cultured both with and without bacteria, we undertook extensive mutational analysis of both the MAIT TCR and MR1 molecule. We found differential contribution of particular amino acids to the MAIT TCR-MR1 interaction based upon the presence of bacteria, supporting the hypothesis that the structure of the MR1 molecules with the microbial-derived ligand(s) differs from the one with the endogenous ligand(s). Furthermore, we demonstrate that microbial-derived ligand(s) is resistant to proteinase K digestion and does not extract with common lipids, suggesting an unexpected class of antigen(s) might be recognized by this unique lymphocyte population.

## Introduction

MAIT cells are a subset of T lymphocytes bearing a semi-invariant αβ TCR that recognize the MHC-related protein 1 (MR1), a class Ib molecule encoded by the non-MHC linked monomorphic *Mr1* gene [1]. MR1 appears unique to mammals and is highly conserved [2], suggesting that it might have evolved under strong selection pressure to fulfill a unique function within the immune system, possibly imposed by immune response to pathogens. MAIT cells are activated by cells infected with various strains of bacteria and yeast in both human and mice [3], [4]. MAIT cells protect mice injected with *E. coli* [3], and MR1-deficient animals have increased bacterial load after *Klebsiella pneumoniae* or *Bacillus Calmette-Guérin* injection compared to controls [5], [6]. In humans, MAIT cells are found at a high-frequency in pools of *M. tuberculosis*-reactive T cell clones [4], and their number in the blood decreases in patients with active bacterial infection [3]. Upon stimulation, mouse MAIT cells produce large amount of diverse cytokines [7], while sorted human MAIT cells, stimulated by anti-CD3 and -CD28 antibodies or *E. coli*-infected cells, rapidly produce IFN-**γ** and TNF-α, as well as granzyme A and B [3], [4]. This capacity to react rapidly to bacterial challenge provides a potential role for the MAIT cells in anti-microbial defense.

These findings have led to the idea that MAIT TCRs react with MR1 bound to a microbe-derived ligand. In addition, certain clones of MAIT cells detect non-infected MR1-expressing APCs, suggesting that, like iNKT cells [8], some MAIT TCRs might have a dual specificity for both microbe-derived antigen(s) as well as APC-derived, or media-provided, antigen(s). Two out of twenty MAIT cell hybridomas were shown to respond to cell lines overexpressing MR1 without exogenous antigen added to the culture media [1], 9. Although all of these hybridomas express the invariant MAIT TCRα chain, they express different TCRβ chains, raising the possibility that each hybridoma might have a different antigenic specificity. Therefore, it is still unclear whether a single MAIT TCR can recognize both bacteria-derived antigen(s) and self-antigen(s) presented by MR1 or whether different subpopulations of MAIT cells are directed against different antigen(s). Furthermore, the contribution of the various CDRs of the mouse MAIT TCR to the recognition of the antigen-MR1 complex remains unknown.

Here we show that the *E. coli*-derived antigen(s) is resistant to proteinase K digestion and lipid extraction and provide evidence that the MAIT TCR uses overlapping but distinct residues for the recognition of MR1 on infected cells and MR1 overexpressing cells in the absence of infection.

## Materials and Methods

### Cell Lines and mAbs

The mouse embryonic fibroblast LM1.8, mouse B cell line CH27, 6C2 MAIT hybridoma and TCRαβ-negative 5KC-78.3.20 hybridoma have been described previously [1], [10]–[12]. All cells were maintained in complete SMEM with 10% fetal calf serum. Anti-MR1 mAb 26.5 has been previously described [13] and was purified in house.

### Modeling of Mouse MR1

Mouse MR1 sequence (UniProt accession number Q8HWB0, http://www.uniprot.org/) was used to model mouse MR1 with the homology-based web server Phyre [14]. The crystal structure of human MR1 [15] was used as a template and mouse MR1 was modeled with 100% confidence. The molecular graphic representation was created with PyMol.

### Proteinase K Digestion and Lipid Extraction

*E. coli* cultures were grown overnight in Luria broth, sonicated, and the <10 kD fraction was separated using Amicon spin columns. Ovalbumin protein (Sigma) was spiked into the fractions containing proteinase K (80 µg/ml), 1 mM CaCl_2_, 50 mM Tris and 10 µM 2-ME. Fractions were incubated at 37°C for 24 hours. After 24 hours, the proteinase K activity was inhibited by adding PMSF (40 mM).

α-Galactosylceramide (Funakoshi co, ltd, Japan) was spiked into overnight *E. coli* cultures and lipids were extracted using the method of Folch [16]. Each phase was dried separately under nitrogen (organic phase) or speedvac (interphase and aqueous). Samples were resuspended in complete media and used to stimulate the indicated hybridoma.

### Hybridoma Stimulation

5×10^4^ hybridomas were cultured for 20 hr with 5×10^4^ APCs transfected or not with mouse MR1, in the presence or not of 20 µg/ml of the blocking anti-MR1 mAb (26.5) or isotype control. Bacterial dilutions were added to antigen presenting cells and hybridomas in complete media with antibiotics. Hybridoma responses were measured by an IL-2 ELISA using standard protocols.

## Results and Discussion

### Alanine-scan Mutagenesis of the 6C2 MAIT TCR

The 6C2 MAIT TCRα and β chains were expressed in a TCR-negative hybridoma. The TCR-expressing hybridoma produced large amounts of IL-2 (10 to 100-fold over background) when co-cultured with LM1.8 fibroblasts transduced with a mouse MR1-encoding construct [10] and the response could be blocked by the addition of anti-MR1 but not isotype control mAbs (data not shown), thereby reproducing the reactivity of the original 6C2 TCR [1].

**Figure 1:**
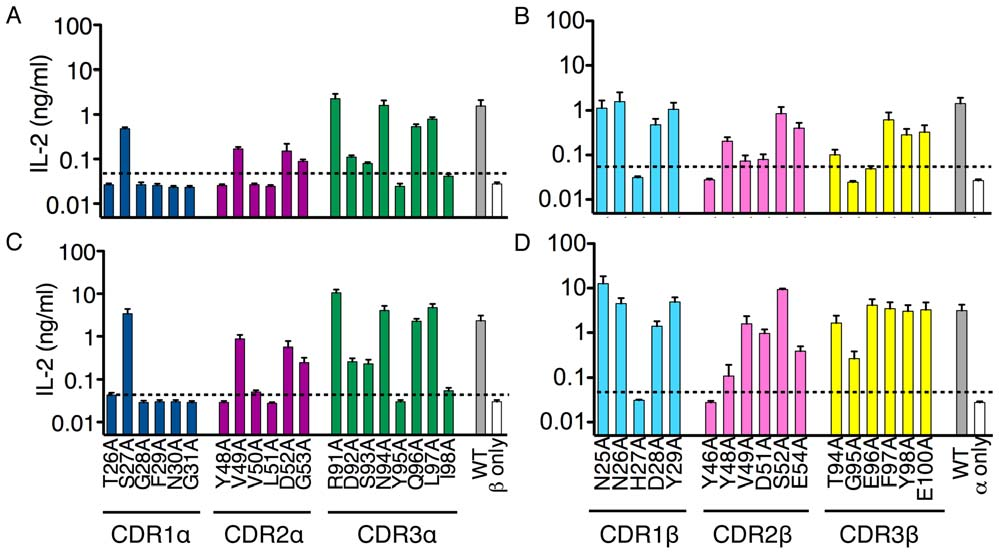
Mutational analysis of the mouse 6C2 MAIT TCR in response to MR1-overexpressing antigen presenting cells. Response of 6C2 MAIT hybridoma expressing different mutations of the TCR α chain (A, C) or TCR β chain (B, D) to overexpression of mouse MR1 on LM1.8 fibroblasts (A and B) or CH27 B cells (C and D). Data represent the mean+s.e.m. of three independent experiments.

Individual residues in the CDRs of the 6C2 TCR were substituted with alanine and each mutant chain was expressed together with the appropriate wild-type partner. Each hybridoma was sorted for similar level of TCR surface expression and the sorted cells of each mutant were demonstrated to have equivalent responses to anti-CD3, anti-CD28-coated plate stimulation (data not shown). Stimulation of these hybridomas using the LM1.8 fibroblast overexpressing MR1 (Fig. 1, A and B) provided us with the pattern of reactivity of these mutants to the presumably self-antigen(s) expressed in fibroblast cells and presented by MR1 molecules or, alternatively, to ligand(s) provided by the culture media. Several interesting observations can be drawn from these results. First, the requirement for residues encoded within the invariant TCRα chain, especially the CDR1α loop, is much more pronounced than it is for residues within the TCRβ chain. Residues within the CDR1α (T26α, G28α, F29α, N30α, G31α), CDR2α (Y48α, V50α, L51α) and CDR3α (D92α, S93α, Y95α, I98α) loops were all necessary for the recognition of the self-antigen-MR1 complex.

Based on the mouse TRAV1 (used by MAIT cells), TRAV11 (used by iNKT cells) and TRAV19 (used by conventional T cells) sequences, we searched databases for orthologue genes in sixteen different species of placental mammals and performed a phylogenic analysis rooted to the TRAV1S1 sequence from *Oncorhynchus mykiss* (Rainbow Trout) as a reference. Based on this analysis, the different mammalian TRAV1 genes appear more closely related to each other than the TRAV11 and TRAV19 genes are, suggesting that TRAV1 genes might have been more conserved in the course of evolution than those of TRAV11 or TRAV19 (Fig. S1A). Sequence alignment showed that all the MAIT TCRα residues involved in the recognition of antigen-MR1 are conserved, thereby providing a potential explanation for the unique use of the TRAV1 and TRAJ33 gene segments by the MAIT TCR (Fig. S1B).

In addition, several residues within the 6C2 Vβ chain affected the recognition of mouse MR1. Mutations of histidine 27 in the CDR1β loop and of Y46β in the CDR2β loop abolished reactivity to MR1 on fibroblasts. Interestingly, tyrosine residues at position 46 and 48 of the CDR2β loop of Vβ8 family gene segments have been proposed as important evolutionarily conserved residues for the generic recognition of MHC molecules [12]. Thus our results potentially extend the involvement of the Y46β residue to the recognition of MR1.

Two residues, G95β and E96β, within the variable CDR3β loop appeared important to the recognition of a putative self antigen(s) presented by MR1 transfected fibroblasts. Although the glycine residue is unlikely to represent a direct contact residue, its flexibility might allow for the CDR3β loop to adopt the “right” configuration necessary for recognition, suggesting the possibility that different MAIT TCRs might use such flexibility to fine-tune their antigen specificities. Further mutational analysis of other autoreactive mouse MAIT TCRs will be necessary to demonstrate whether these results are restricted to the 6C2 TCR or can be extended the MAIT population in general.

Overall, the majority of residues important for recognition of self-MR1 in the MAIT TCR are concentrated in the TCRα chain, however, the TCRβ chain contributes a significant portion of these residues as well. This supports the idea that the degree of conservation of the MAIT TCRα chain is important for reactivity to MR1 and the usage of several different TCRβ chains might allow for more flexibility. These results are in agreement with a recent study that identified the energetically important residues for the recognition of MR1 by the human MAIT TCR with the exception that no residue within the human Vβ chain was found essential in mediating MR1-restricted activation of the human MAIT TCR [17].

### MAIT TCR Mutations Reveal a Similar Pattern of Reactivity to MR1-transfected B cells

The ubiquitous expression of MR1 transcripts suggests that several cell types might be able to present antigens to MAIT cells. However, it remains unclear whether MR1 might be presenting antigen(s) common to all APCs or whether different APCs might each express a unique set of antigen(s). In support of the latter possibility, B cells are uniquely required for the expansion of MAIT cells in the periphery [1], [18].

We compared the reactivity of each of our MAIT TCR mutants with the LM1.8 fibroblast and the CH27 B cells, which overexpress MR1 on their surface. The pattern of responses of the hybridoma collection to B cells was similar to that obtained with fibroblasts (Fig. 1, C and D). Minor differences between the two APC types were noticed for residues V49β, D51β and E96β but they probably reflect the stimulatory effectiveness of each APC than actual differences in MR1 recognition. Indeed, rat MR1-transfected CH27 cells, which provide a stronger stimulatory signal to the 6C2 hybridoma [19], did not perturb the pattern of recognition of the different TCR mutants (data not shown).

Thus, the self antigen(s) presented by MR1 expressed on cell lines of two different origins (fibroblast and B cells) appear to be similar in their ability to engage a MAIT TCR.

### MAIT TCR Mutants Reveal a Different Pattern of Reactivity to Cells Co-cultured with E. coli

Various strains of bacteria and yeast activate MAIT cells in an MR1-dependent manner [3], [4]. A still unidentified antigen(s) common to these microbes is thought to be directly presented by MR1 to MAIT cells. The 6C2 hybridoma responded to MR1 transfectants but was unresponsive to untransfected APCs. Addition of *E. coli* to the cultures induced a response by the hybridoma when added to the untransfected APCs while it only minimally increased the autoreactive response (Fig. S2). MR1 mRNA and protein are ubiquitously expressed, including in the untransfected APC cell line used [11], but the cell surface expression of endogenous MR1 is extremely low and is not sufficient to readily trigger a response [20]. Addition of *E. coli* to the hybridoma in absence of APCs also does not trigger any IL-2 release from the hybridoma suggesting that the observed response is not due to autopresentation. Yet, the hybridoma response can be blocked by the addition of anti-MR1 mAbs when *E. coli* is added to the untransfected line. These results suggest that addition of *E. coli* might induce endogenous MR1 to accumulate on the surface of APCs at levels that are sufficient to activate MAIT cells. However, these expression levels remain too low to be detected serologically ([20] and data not shown).

Bacteria could induce the response either by directly providing a product or by indirectly activating the production of a self-ligand that, when bound to MR1, engages the TCR and activates MAIT cells. The former idea is likely correct since previous studies have indicated that MAIT cell activation occurs independently of Toll-like receptors or other innate pathways and that MAIT cells can be activated by fixed APCs cultured in the presence of bacteria [3].

**Figure 2:**
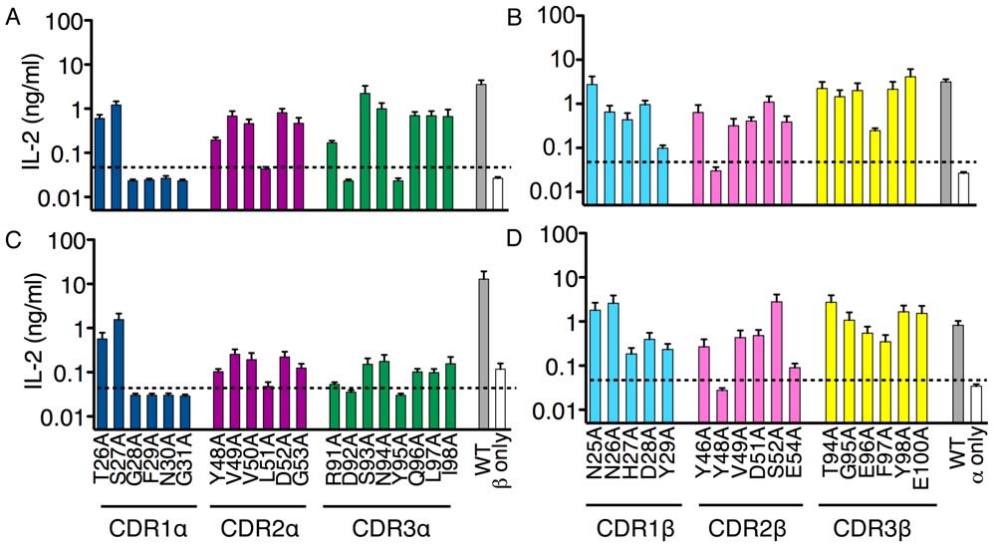
Mutational analysis of the mouse 6C2 MAIT TCR in response to antigen presenting cells infected with *E. coli*. Response of 6C2 MAIT hybridoma expressing different mutations of the TCR α chain (A, C) or TCR β chain (B, D) to LM1.8 fibroblasts (A and B) or CH27 B cells (C and D) cocultured with *E. coli* (MOI = 50). Data represent the mean+s.e.m. of three independent experiments.

Several mutations in both the TCRα and TCRβ chains had different effects depending whether the response was directed against self or the bacteria-induced response (Fig. 2A and B). In the TCRα chain, alanine substitution of T26α in the CDR1α and Y48α, V50α, S93α and I98α in the CDR2α and CDR3α loops affected the autoreactivity but not the bacteria-induced response. In contrast, mutation of R91α in CDR3α had the opposite affect since it did not influence the autoreactive response but decreased the bacteria-induced response. For the TCRβ chain, H27β, Y46β, and G95β were differentially required between the autoreactive and bacteria-induced responses since alanine substitution of these residues resulted in loss of autoreactivity, but had no effect on the *E. coli*-induced response.

The differential sensitivity to TCR sequence changes of the two responses could be detected using either fibroblasts or B cells as APCs, although overall the response of the mutants to the B cells was lower (Fig. 2C and D). Altogether, these results suggest that an *E. coli*-derived antigen(s) is directly recognized by the MAIT TCR in a manner that is similar but not identical to the recognition of the self-antigen(s). It is tempting to speculate that different antigen(s) might be recognized in the two situations or that the bacteria-derived antigen induces MR1 to adopt a slightly different conformation than the self-antigen(s).

### Effect of MR1 Mutations on the Reactivity of the 6C2 MAIT TCR

**Figure 3:**
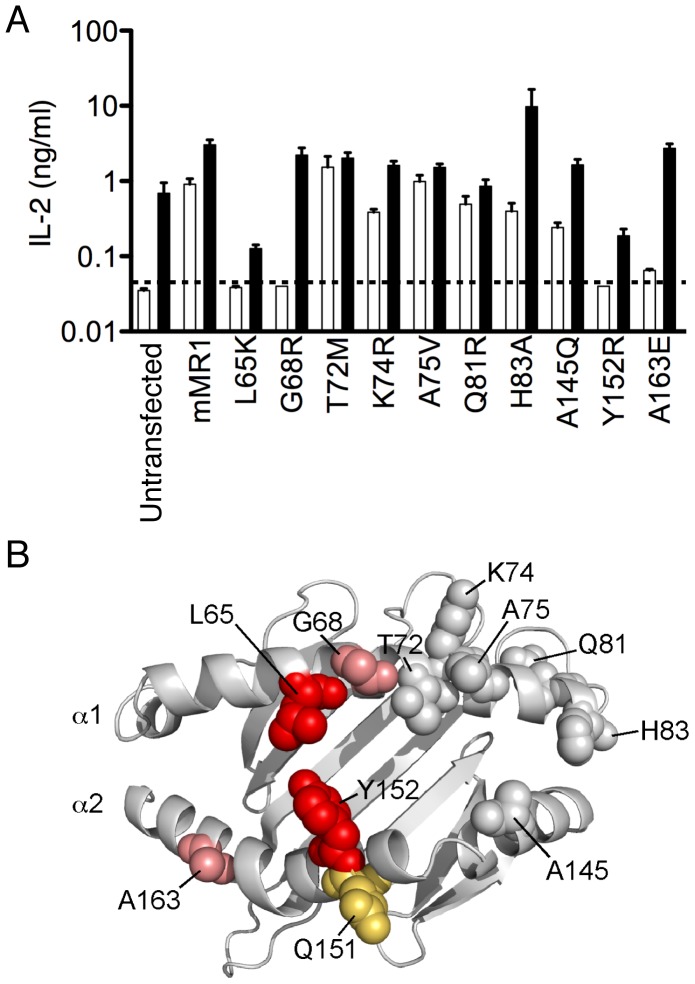
The MAIT hybridoma response to MR1 mutants. (A) The 6C2 MAIT hybridoma was stimulated with mouse MR1 mutants expressed on LM1.8 fibroblasts in the absence (white bars) or presence of bacteria (black bars) at an MOI of 100. The IL-2 response in both cases could be blocked with an anti-MR1 antibody (data not shown). (B) The effect of the MR1 mutations on both the autoreactive response and the bacterial response is mapped on Phyre-generated mouse MR1 model. Mutations with an effect on both the autoreactive and bacterial response are shown in red, while mutations that only affected the autoreactive response are shown in pink. The residue attributed to the inability of human MR1 to activate mouse MAIT [19] cells is shown in orange.

Next, we analyzed how 10 different mutations localized in the predicted helical residues of the MR1 molecule [10] affected the response of the 6C2 hybridoma to MR1-overexpressing APCs or its response to the *E coli*-derived ligand(s). Two mutations located on the α1 helix (L65 and G68) and two mutations on the α2 helix of MR1 (A163 and Y152) affected the autoreactivity of the 6C2 hybridoma (Fig. 3A). Only the residues L65 and Y152 remained essential to the reactivity of the 6C2 TCR towards the *E. coli*-derived ligand. These centrally located residues (Fig. 3B), demonstrate an extremely focused energetic hot spot on the MR1 molecule necessary for the recognition by the 6C2 TCR. Interestingly, this hot spot is located next to the Q151 residue. Such position is encoded by a leucine in human MR1 and was shown to be the sole reason that human MR1 fails to activate mouse MAIT cells [19].

### The E. coli-derived MAIT Antigen is Resistant to Proteinase K Digestion and Lipid Extraction

The nature of the antigen(s) recognized by MAIT cells remains to be determined. Previous experiments have suggested that it was sensitive to protease digestion [4]. Glycolipids have also been proposed as MAIT cell antigens [21].

A <10 kD fraction isolated from *E. coli* contains the activity that stimulates the 6C2 hybridoma but not the OVA-specific DO11.10 hybridoma or the α-Galcer-reactive DN32.D3 hybridoma (data not shown). We therefore independently spiked the fraction with either ovalbumin or α-Galcer.

**Figure 4:**
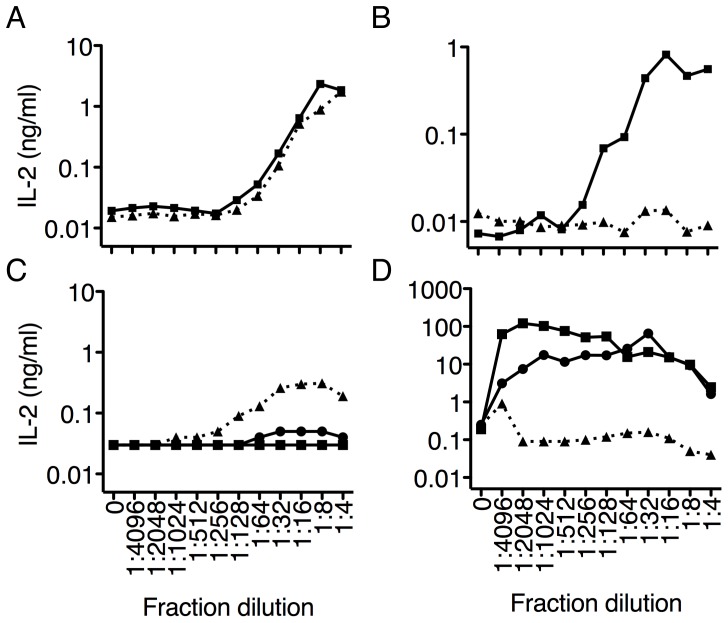
The MAIT ligand is resistant to digestion with proteinase K and is not part of the lipid component of <10 kD *E. coli* culture. The <10 kD fraction *E. coli* lysate was spiked with OVA protein and left untreated (solid line) or digested overnight with proteinase K (dashed line). MAIT ligand activity was tested with LM1.8 fibroblasts and the 6C2 hybridoma (A) while OVA antigenic activity was tested with A20 cells and the DO11.10 hybridoma (B). An overnight culture of *E. coli* was spun down, resuspended in chloroform/methanol, spiked with α-GalCer, sonicated and subjected to lipid extraction. Aqueous (triangles), interphase (circles), and organic (squares) phases were dried out and resuspended in complete media. MAIT ligand activity was assessed with the 6C2 hybridoma and LM1.8 fibroblasts at indicated dilutions (C) while α-GalCer activity was detected using the DN32.D3 hybridoma and mCD1d-transfected A20 cells at indicated dilutions (D). Data represent the mean+s.e.m. of two independent experiments.

The fraction was further treated, or not, with proteinase K and used to individually stimulate the DO11.10 or the MAIT 6C2 hybridoma. Proteinase K treatment of the OVA-spiked fraction completely abrogated the response of the DO11.10 hybridoma (Fig. 4B), while the stimulation of the 6C2 hybridoma was unaffected (Fig. 4A).

Alternatively, the fraction was extracted with the Folch method to separate polar from non-polar compounds [16]. The organic, interphase and aqueous phases were isolated, dried and resuspended in complete media. Each phase was then used to stimulate the 6C2 or DN32.D3 hybridomas. As expected, αGC activity was found within the organic and interphases but was absent from the aqueous phase (Fig. 4D). In sharp contrast, only the aqueous phase contained the stimulatory activity for the MAIT 6C2 hybridoma (Fig. 4C). Altogether, these data show that the MAIT ligand is rather hydrophilic but is resistant to proteinase K digestion. These characteristics contrast with the nature of most of the currently known antigens presented to T cells (ie. peptides by conventional MHC class I and II, formylated-peptides by H2-M3, phospholipids, glycolipids and lipopeptides by members of the CD1 family), and argue that MAIT cells might be directed at an unusual class of antigen(s).

While our manuscript was under review, Kjer-Nielsen and colleagues [15] reported the first crystal structure of human MR1 in complex with 6-formyl pterin, a vitamin B9 metabolite. Further, they showed that other metabolites originating from the riboflavin (vitamin B2) metabolic pathway could stimulate human MAIT cells in the presence of an Epstein-Barr virus (EBV)-transformed B cell lymphoblast transfected with a human-MR1-encoding construct [15]. These results are in good agreement with the findings described above and suggest that vitamin B metabolites might represent a new class of antigens that are presented by MR1 for MAIT-cell immunosurveillance of microbial infections.

## Supporting Information

Figure S1 Comparison of orthologous Vα19-Jα33 gene segments in several species that express MR1. (A) Rooted phylogenic tree of TRAV1, TRAV11 and TRAV19 orthologues from 16 different mammalian species. (B) Sequence alignment of TRAV1 and TRAJ33 orthologues from 16 different mammalian species. Conserved cysteine residues important to the immunoglobulin fold are highlighted in red, while residues important for recognition of antigen-MR1 complex are in bold.
(TIFF)

Figure S2 Response of 6C2 MAIT hybridoma to fibroblasts overexpressing mouse MR1 (hatched bars) or untransduced fibroblasts (gray bars) cocultured with *E. coli* at indicated MOI. MR1 blocking antibody 26.5 (20 µg/mL) was used to inhibit the response of the hybridoma to wild-type fibroblasts (black bars) co-cultured with *E. coli*. ELISA of IL-2 production by hybridoma following overnight culture with indicated APCs was determined by ELISA. Data represent the mean+s.e.m. of three independent experiments.
(TIFF)
