# Supplementary figures and images for: MAIT Cell Recognition of MR1 on Bacterially Infected and Uninfected Cells
Source: PLoS One. 2013 Jan 14;8(1):e53789. doi: 10.1371/journal.pone.0053789 (PMC3544856; doi:10.1371/journal.pone.0053789)

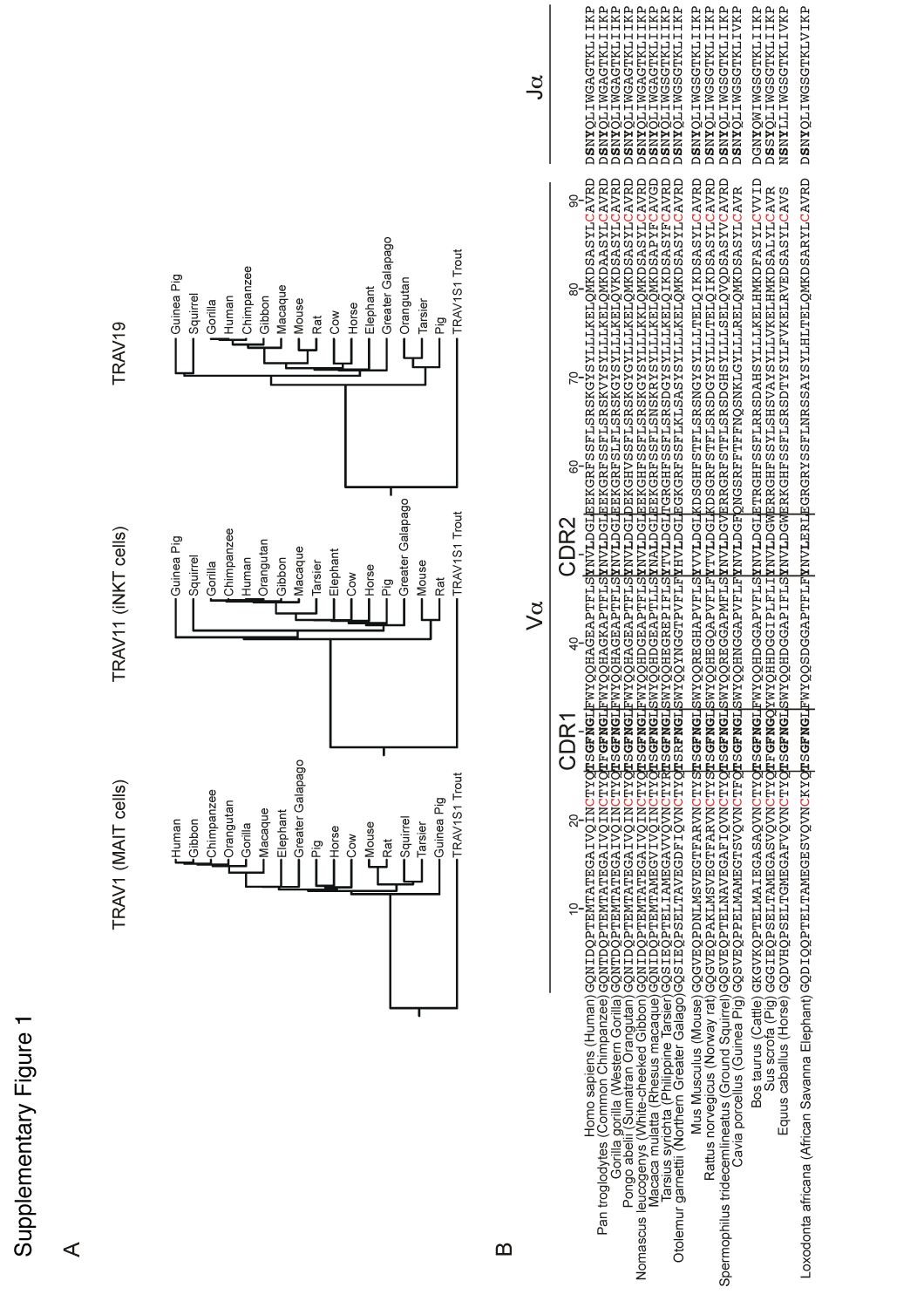

Supplement: Figure S1 — Comparison of orthologous Vα19-Jα33 gene segments in several species that express MR1. (A) Rooted phylogenic tree of TRAV1, TRAV11 and TRAV19 orthologues from 16 different mammalian species. (B) Sequence alignment of TRAV1 and TRAJ33 orthologues from 16 different mammalian species. Conserved cysteine residues important to the immunoglobulin fold are highlighted in red, while residues important for recognition of antigen-MR1 complex are in bold. (TIFF) [file pone.0053789.s001.tiff]

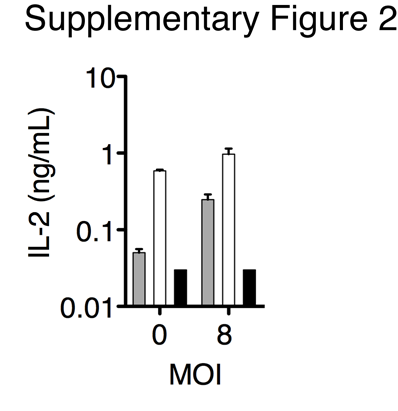

Supplement: Figure S2 — Response of 6C2 MAIT hybridoma to fibroblasts overexpressing mouse MR1 (hatched bars) or untransduced fibroblasts (gray bars) cocultured with E. coli at indicated MOI. MR1 blocking antibody 26.5 (20 µg/mL) was used to inhibit the response of the hybridoma to wild-type fibroblasts (black bars) co-cultured with E. coli. ELISA of IL-2 production by hybridoma following overnight culture with indicated APCs was determined by ELISA. Data represent the mean+s.e.m. of three independent experiments. (TIFF) [file pone.0053789.s002.tiff]
